# Phylogeography of *Heteropriacanthus*, a circumtropical reef fish with very little morphological variation

**DOI:** 10.1038/s41598-026-47623-2

**Published:** 2026-04-11

**Authors:** H. A. Lessios, A. Calderón, L. Calderon, L. B. Geyer

**Affiliations:** Smithsonian Tropical Research Institute, Balboa, Panama

**Keywords:** Cosmopolitan species, Marine phylogeography, Stepping stone model, Isolation by distance, Ecology, Ecology, Evolution, Genetics, Ocean sciences, Zoology

## Abstract

Only six species of fishes found on reefs are known to have a circumtropical distribution. Until recently *Heteropriacanthus cruentatus* was one of the six, recognized as a single species in a monotypic genus present in all tropical and subtropical seas. Based on sequences of cytochrome oxidase I and on morphology, a previous study split the genus into three species, *H. carolinus* in the Indo-Pacific, *H. cruentatus* in most of the tropical Atlantic, and *H. fulgens* in the northeast Atlantic. We sampled two mitochondrial and three nuclear genes (a total of 3618 bases) from 21 locations in three oceans to determine the phylogeography and genetic structure of the genus. We found three clades, corresponding to *H. cruentatus*, *H. fulgens*, and *H. carolinus*. *H. fulgens* extends, not just to the northeastern Atlantic as previously known, but also to the southeast, the central and (possibly) the southwest Atlantic, whereas *H. cruentatus* is restricted to the Greater Caribbean. *H. carolinus* spreads in the entire Indo-Pacific. We found no evidence that the three species are sympatric anywhere in their range. *H. cruentatus* is genetically homogeneous over its small range, but *H. fulgens* shows evidence of gene flow restrictions between the northeast and the southeast Atlantic and between the east and the central Atlantic. Genetic exchange in *H. carolinus* is restricted by the large expanse of open water between the eastern and the central Pacific (the 4000 km wide Eastern Pacific Barrier), and by either distance or historical separation between the Pacific and the Indian Oceans. There is evidence of isolation by distance in *H. carolinus*, but not in *H. fulgens* or *H. cruentatus*. A coalescent Bayesian Analysis also supported a stepping stone model in *H. carolinus* and indicated that gene flow through the Eastern Pacific Barrier was mostly eastwards, whereas it was westwards between the central and western Pacific. Genetic exchange was high and symmetrical between the western Pacific and the Indian Ocean. Thus, despite the remarkably low morphological variation of *Heteropriacanthus*, its phylogeography shows that gene flow is restricted by some of the major barriers to dispersal for marine organisms. Nevertheless, the observed degree of connectivity across the width of the Indo-Pacific is remarkable for a reef fish.

**Supplementary Information:**

The online version contains supplementary material available at 10.1038/s41598-026-47623-2.

## Introduction

Marine species thought to have circumtropical distributions, because they are morphologically homogeneous, are obvious targets for genetic studies. Such studies aim to determine whether the assignment of geographically distant but morphologically similar populations to the same species is the mark of cryptic speciation. In many cases molecular data have shown that populations from different oceans belong to different lineages, suggestive of cryptic species (reviews in^1–4^). Often the revealed cryptic species are distributed on either side of one or more of the major barriers that separate oceanic regions^5–7^. The interruption of the Tethys Sea, the Central American Isthmus, and the Benguela upwelling off the southwest coast of Africa isolate the tropical and subtropical zones of the Atlantic from those of the Indo-Pacific. The 4000 km stretch of open ocean with no stepping stones between the coast of America and the central Pacific (the East Pacific Barrier) isolates biota of the eastern Pacific from the rest of the Pacific. Open water also stretches between the shores of the east and west Atlantic, with only the isolated islands of Ascension and St. Helena and the St. Peter and St. Paul Rocks in the central Atlantic. The fresh water plumes and sediment outflow of the Orinoco and Amazon Rivers constitute a salinity and habitat barrier between the Caribbean and the Brazilian coast. A historical barrier between the western Pacific and Indian Ocean was the Pleistocene emergence of the Sunda Shelf.

Only six species of bony fishes found on reefs are known to be circumtropical (*Heteropriacanthus cruentatus, Cookeolus japonicus, Melichthys niger, Chilomycterus reticulatus, Diodon holocanthus*, and *D. hystrix*)^3^. The “glass eye” *Heteropriacanthus cruentatus* was until recently thought to be one of the six. Although *H. carolinus* had been described from the Caroline Islands^8^ and *H. fulgens* from Madeira^9^, they were placed in synonymy^10,11^, and only *H. cruentatus* was thought to be present in all tropical oceans. Starnes^11^, finding no consistent diagnostic characters between *H. cruentatus* and the other two species, determined that *Heteropriacanthus* was a monotypic genus. Lessios^12^, however, reported genetic distances between Atlantic and Pacific *H. cruentatus* populations in ATP synthetase 8 and 6 of 25.2% and 20.9%, five times greater than the average of other fishes that were presumably separated by the Isthmus of Panama but were recognized as separate species. Gaither et al.^13^ found a difference of 10.4% in cytochrome c oxidase I (COI) and a single fixed base pair (bp) difference in S7 ribosomal protein between Pacific and Atlantic populations. Fernandez-Silva and Ho^14^ constructed the phylogeny of a 562 bp fragment of COI of specimens from various localities and found well-supported clades corresponding to the Indo-Pacific, the Caribbean and the northeast Atlantic. This finding, together with observations of small differences in morphology of specimens from the three regions, led them to revive *H. carolinus* and *H. fulgens* as valid species and thus remove *H. cruentatus* from the list of circumglobally distributed species.

*Heteropriacanthus* is a carnivorous fish of bright red and silver coloration, associated with coral and rocky reefs growing to about 51 cm^15^. During the day it is typically found in caves and crevices. At night it is active feeding on fish and invertebrates, a diurnal pattern related to its large eyes^16^. Its bathymetrical distribution ranges from 5 to 432 m. It has planktonic eggs, larvae, and juveniles. The mean planktonic larval duration (PLD) of *H. cruentatus* is 33 days^17^.

We sampled two mitochondrial and three nuclear genes in specimens from the Indo-Pacific and the Atlantic Oceans to reconstruct the phylogeography of *Heteropriacanthus*. We use our data to address the questions: (1) Does the sample of additional genes support the findings of Fernandez-Silva and Ho^14^ regarding the existence of three species? (2) If so, what is the geographical range of each species? (3) What are the barriers that separated the species? (4) What is the genetic structure of each species?

## Materials and methods

**Fig. 1:**
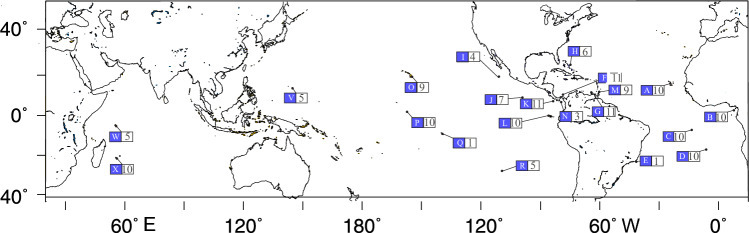
Collection localities and sample sizes of Heteropriacanthus. Box with blue background indicates locality code, open box sample size. Locality codes: **A**: **B**ranco Island, Cabo Verde; **B**: São Tomé; **C**: Ascension; **D**: St. Helena; **E**: Cabo Frio, Brazil; **F**: San Blas and Portobelo, Atlantic Panama; **G**: Los Roques, Venezuela; **H**: Bahamas; **I**: Revillagigedos; **J**: Clipperton; **K**: Isla del Coco, Costa Rica; **L**: Isla Marchena, Galápagos; **M**: Grenada; **N**: Gulf of Chiriqui and Bay of Panama; **O:** Hawaii; **P:** Kiribati, Kiritimati; **Q:** Marquesas; **R**: Easter Island; **V**:Guam; **W**: Seychelles; **X**: Réunion*.*

We collected a total of 154 samples of *Heteropriacanthus* at 21 locations of the Atlantic, Pacific and Indian Oceans (Fig. 1). One specimen of *Priacanthus nasca* from Easter Island served as an outgroup. One specimen from *P. meeki* from Hawaii and two specimens of *P. arenatus* from the Bahamas were also included to verify the monophyly of *Heteropriacanthus*.

Genomic DNA was extracted from gill or muscle either with direct proteinase K digestion^18^ or with a Cetyltrimethylammonium Bromide extraction (CTAB)^19^. We amplified 910 bps of mitochondrial ATP synthetase 8 and 6 (hence ATPase) of 153 *Heteropriacanthus* with the methods of Lessios and Robertson^20^. 1387 bps of the nuclear Recombination Activating Factor1 (RAG1) gene of 130 individuals were amplified using primers RAG1F 5’AGC TGT AGT CAG TAY CAC AAR ATG and RAG9R 5’GTG TAG AGC CAG TGR TGY TT, or HC-Rag1-F2 5’AGT GGC CGC CAG ATC TTT CA and HC-Rag1-R2 5’ TTC ATC TTC CGG AAA CGC CTG AAC. 0.2-0.5 µL of DNA template were amplified in one of two mixes: Mix 1: 50 µL total volume containing 1U/µL of Master Amp *Tfl* polymerase (Epicentre®), 1.25 µL of 10x *Tfl* PCR buffer, 1.25 µL of 25 mM MgCl_2_, 1.25 µL of 8 mM dNTPs, and 0.625 µL of each primer. Mix 2: 50 µL total volume containing 5U/µL of Promega GoTaq Flexi DNA Polymerase^®^, 2.5 µL of 5x Colorless buffer, 1.25 µL 25mM MgCl_2_, 1.25 µL 8 mM dNTPs, and 0.625 µL of each primer. The amplification mix was subjected to initial heating at 94°C for 5 min, then to three cycles of denaturation at 94°C for 40s, primer annealing at 63°C for 60s, extension at 72°C for 60s, then three cycles at 94°C for 40s, 60°C for 60s, 72°C for 60s, then 31 cycles at 94°C for 40s, 55°C for 60s, 72°C for 60s, ending with 5 min at 72°C. 756 bps of Recombination Activating Factor2 (RAG2) from 143 individuals were amplified using primers RAG2-F1 5’GAG GGC CAT CTC CTT CTC CAA and RAG2-R3 5’GAT GGC CTT CCC TCT GTG GGT AC. The same amplification mixes as that of RAG1 were subjected to initial heating at 94°C for 2 min, then eight cycles at 94°C for 45s, 53°C for 45s, 72°C for 55s, then 28 cycles at 94°C for 45s, 53°C for 45s, 72°C for 105s, ending with 5 min at 72°C. 565 bps of TMO-4C4 (TMO) in 149 individuals were amplified with primers TMO-F1 5’CCT CCG GCC TTC CTA AAA CCT CTC and TMO-R1 5’CAT CGT GCT CCT GGG TGA CAA AGT. The same PCR mixes as those of RAG1 were amplified in an initial cycle at 96°C for 5s, then 39 cycles at 94°C for 30s, 50°C for 45s, 72°C for 60s, then one cycle at 72°C for 5 min, ending with 5 min at 10°C.

Bands of the correct size of the amplification products, visualized in agarose gels, were cut from the gels, treated with Gelase, or cleaned with ExoSap. They were cycle-sequenced with the amplification primers (also internal primers RAG5R 5’TRG AGT CAC ACA GAC TGC AGA, and RAG8R 5’CGC CAC ACA GGY TTC ATC T in the case of RAG1) and sequenced in both directions in Applied Biosystems automatic sequencers.

The unique sequences of each DNA region were aligned in MAFFT v. 7.520^21^ and imported in MEGA v.11.013^22^ to determine the simplest model of DNA evolution that produced the best fit of the data to the tree, based on the Akaike^23^ criterion. For ATPase this was the model of Tamura and Nei^24^ with a Γ distribution (α = 0.78) of rates and a proportion of 0.3 of invariable sites. For RAG1 the best model was also that of Tamura and Nei with a Γ distribution (α = 0.05), for RAG2, the model of Tamura^25^ with a Γ distribution (α = 0.0.5.), and for TMO the model of Hasegawa et al.^26^ with a Γ distribution (α = 0.05).

The sequences of the four genes were concatenated for further analysis. Sites of a gene that did not amplify for a particular individual were treated as missing. Nucleotide ambiguities that could be due to heterozygosity in the nuclear genes or to sequencing error were treated as ambiguities. Only two individuals (at Ascension) shared the same concatenated haplotype (Supplementary Table S1). Phylogenies of the concatenated data, applying the best model of evolution to the partition of each gene, were estimated by Bayesian inference in MrBayes v.3.2.7^27^ and by Maximum Likelihood in RAxML-HPC v.8.2.12^28^. In MrBayes we used Dirichlet priors for rates and nucleotide frequencies and fixed values for the gamma distribution of rates and invariable sites. Runs had 8 chains and a heating value of T = 0.01. They consisted of 2x10^6^ steps, sampling every 100th tree and calculating credibility values of the 50% majority tree after discarding the first 5x10^3^ trees. Convergence was determined by a value of average standard deviation of split frequencies <0.01, by a value of potential reduction factor equal to 1 for all parameters, and by multiple runs that produced the same topology. RAxML was run in version 2.0 of the graphical interface of Edler et al.^29^ using “rapid bootstrap” with the same partitions and parameters as those in Mr. Bayes. Median-joining networks^30^ were constructed in POPART^31^. As the long concatenated sequences resulted in a set of unique genotypes (except for two individuals in Ascension), the networks were constructed only for the mitochondrial genes. Maximum Composite Likelihood Genetic distances (MCL)^32^ were calculated in MEGA v.11.0.13^22^.

Φ_ST_ values and Analysis of Molecular Variance (AMOVA) were calculated in ARLEQUIN v.3.5.2.2^33^. Their significance was determined by comparison to 1023 random reshufflings of alleles between populations. The hierarchical levels of this analyses were populations and biogeographic regions, defined as the eastern, central and western Atlantic, or the eastern, central and western Pacific, and the Indian Ocean. Significance of Φ_ST_ values was adjusted for multiple comparisons with the modified false discovery rate procedure^34^ as recommended by Narum^35^. Isolation by distance analysis was conducted with IBD v.1.52^36^, to test for Mantel^37^ correlations between Φ_ST_ values and geographic distance by sea. Given the large geographic distances involved, distance data were converted to their base 10 logarithms.

To test alternative models of gene flow between populations and to determine the direction of genetic exchange, we used MIGRATE-n v. 5.0.6^38^, a coalescent Bayesian approach considered as more informative than F_ST_ statistics^39^. MIGRATE-n estimates marginal likelihoods to be used in Bayes Factor comparisons between models, so that the one to which the data have the best fit can be chosen. It also provides estimates of migration between populations in each direction, information that cannot be obtained from F_ST_ statistics, which assume symmetrical flow. The models we tested against each other in the Atlantic and the Indo-Pacific were different, according to the hypotheses we considered as supported by geography and phylogeny. For the Atlantic populations, we compared models that assumed: (1) Panmixia, i.e. random mating in the entire ocean. (2) Wright’s^40^ island model, according to which each biogeographical region is exchanging genes in both directions with each other region. (3) A model that stipulated that the western Atlantic is one population, and the combined central and eastern Atlantic is another. For the Indo-Pacific, we tested Wright’s island model against Kimura and Weiss’s stepping stone model^41^, limiting genetic exchange only between neighboring regions. The probability of the models relative to each other was determined from Bayes Factors estimated from marginal likelihood Bezier-approximated through thermodynamic integration^42^. The program was run with the appropriate inheritance multipliers for each locus (0.25 for ATPase, 1.00 for the three nuclear loci), allowing it to estimate mutation rates of each from the data. Uniform priors were used for Θ (the product of mutation rate times effective population size) and for migration rate M. The program was run with four MCMC chains of 10^7^ steps (heating scheme 1, 1.5, 3 and 10^6^), recording every 100^th^ step. Convergence was determined by comparing the marginal likelihoods produced in multiple runs. The mean likelihoods over all loci of multiple runs were averaged in the calculation of Bayes Factors and then used to determine model probabilities.

## Results

**Fig. 2:**
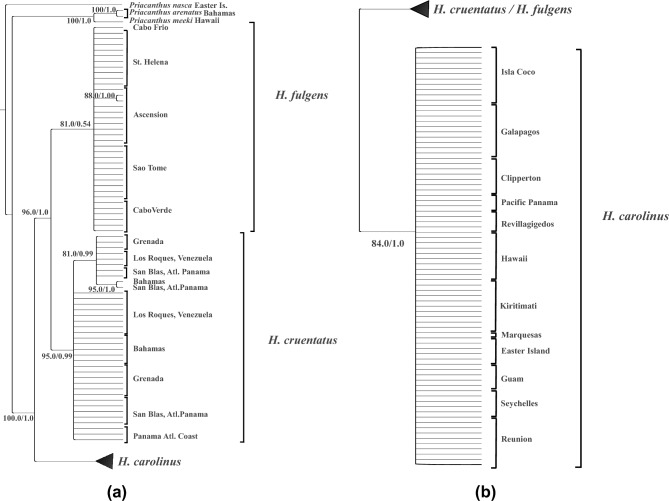
**A**: First part of phylogenetic tree of *Heteropriacanthus* based on 910 bps of ATP synthetase 8 and 6, 1387 bp of Recombination Activating Factor 1, 756 bps of Recombination Factor 2, and 565 bps of TMO-4C4, reconstructed with MRBAYES^27^ and RAxML^28^. Support of nodes from bootstrapping in RAxML is followed by posterior probabilities from MRBAYES. **B**: Second part of phylogenetic tree of *Heteropriacanthus.*

**Fig. 3:**
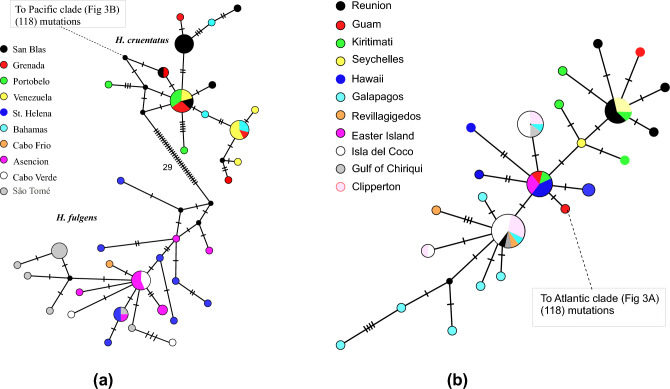
**A**: First part of Median-joining network of haplotypes of ATP synthetase 8 and 6 of *Heteropriacanthus.* Each colored circle shows a unique haplotype; small black circles represent hypothetical, unsamspled haplotypes. Different colors indicate localities; the diameter of each circle is proportional to the number of individuals with that haplotype. Tick marks or numbers represent the number of substitutions between haplotypes. **B: **Second part of Median-joining network of haplotypes of ATPase 8 and 6 of *Heteropriacanthus.*ΦST statistics are an indication of differentiation between populations relative to variation within populations.

The phylogeny of the species of *Heteropriacanthus* is shown in Figs. 2A and 2B. The concatenated sequences of ATPase8 and 6, RAG1, RAG2, and TMO (3618 bps) formed deeply separated clades (MCL distance of 5.35%) between those from the Atlantic (*H. cruentatus* + *H. fulgens*) and those from the Pacific (*H. carolinus*) Oceans. Within *H. carolinus*, there was no phylogenetic distinction between sequences from any Pacific region, nor between those from the Pacific and those from the Indian Ocean, despite a geographic distance of 25,000 km between Panama and Réunion (Figs. 2B and 3B).

In the Atlantic, the data suggested that *H. fulgens* and *H. cruentatus* are distinct (MCL distance of 1.42%), although the monophyly of *H. fulgens* was only moderately supported by RAxML and weakly supported by MRBAYES. Separate phylogenies of each gene (Supplemental Figs. S1-3) indicated that nearly all variation contributing to the separation of the clades comes from the mitochondrial genes (MCL distance of 5.34%) with no individuals with identical haplotypes between the two species (Fig 3A), whereas the nuclear ones, despite the long sequences, failed to form separate clades. Sequences of *H. fulgens*, thus far described only from the northeastern Atlantic^14^, were present in southeastern (São Tomé) and the and central (Ascension and St. Helena) Atlantic locations, without any phylogenetic structure between sequences from the two regions. The single individual that we were able to collect from Cabo Frio, Brazil turned out to belong to *H. fulgens* instead of *H. cruentatus*. Its ATPase sequence differs by only one substitution from the most common haplotype in Ascencion and Cabo Verde (Fig. 3A). We found no sequences of *H. cruentatus* outside the Greater Caribbean. A subclade within *H. cruentatus* is well supported but shows no phylogeographic structure because it is composed of sequences from four locations where sequences outside this clade are also found.

Φ_ST_ values indicated that there was little difference within each major oceanic region in the Indo-Pacific. Values between populations of *Heteropriacanthus carolinus* within the Indian Ocean, the central, or the eastern Pacific were mostly small and not significantly different from random (Table 1). The only population we sampled in the western Pacific, Guam, is also not significantly different from any other population, except Réunion. There was, however, differentiation between populations from different regions. Φ_ST_ values between populations in the Indian Ocean, on the one hand, and those of the central (except for Kiritimati) and eastern Pacific, on the other, were large and significant. As the result, there was a positive correlation between Φ_ST_ and the logarithm of geographic distance (r = 0.243, p < 0.05). AMOVA analysis showed that in *Heteropriacanthus carolinus* most variation was within each population but that Φ_ST_ values between populations and between regions were also large and significantly different from random (Table 2).

**Table 1:** ΦST statistics comparing populations of each species of *Heteropriacanthus* in which more than 5 sequences were obtained. Significance was determined by comparison to 1023 random reshufflings of sequences between populations. NS: not significant, *: p<0.05 after correction for false discovery rate.

*H. cruentatus*																					
	Atlantic_Panama		Venezuela		Grenada		Bahamas														
Atlantic_Panama	-																				
Venezuela	0.14	*	-																		
Grenada	0.06	NS	0.12	NS	-																
Bahamas	0.11	NS	-0.01	NS	0.08	NS	-														
*H. fulgens*	Central Atlantic				East Atlantic																
	Ascension		St_Helena		Cabo_Verde		Sao_Tome														
Ascension	-																				
St_Helena	0.11	NS	-																		
Cabo_Verde	0.39	*	0.38	*	-																
Sao_Tome	0.22	*	0.30	*	0.44	*	-														
*H. carolinus*	Indian Ocean				W. Pacific		Central Pacific						Eastern Pacific								
	Seychelles		Reunion		Guam		Easter_Is		Hawaii		Kiritimati		Revillagigedo		Pac. Panama		Is_Coco		Galapagos		Clipperton
Seychelles	-																				
Reunion	0.11	NS	-																		
Guam	0.31	NS	0.37	*	-																
Easter_Is	0.47	*	0.53	*	-0.14	NS	-														
Hawaii	0.41	*	0.45	*	0.09	NS	0.16	NS	-												
Kiritimati	0.04	NS	0.15	NS	-0.02	NS	-0.04	NS	0.14	NS	-		-								
Revillagigedo	0.39	*	0.47	*	0.06	NS	0.15	NS	0.22	NS	0.25	*	-								
Pacific Panama	0.53	NS	0.55	*	0.07	NS	0.48	NS	0.29	*	0.14	NS	-0.34	NS	-						
Is_Coco	0.63	*	0.58	*	0.16	NS	0.56	*	0.37	*	0.24	*	-0.22	NS	0.01	NS	-				
Galapagos	0.38	*	0.39	*	0.15	NS	0.23	*	0.25	*	0.24	*	0.01	NS	-0.13	NS	-0.01	NS	-		
Clipperton	0.53	*	0.53	*	0.06	NS	0.39	*	0.28	*	0.17	NS	-0.28	NS	-0.05	NS	0.02	NS	-0.09	NS	-

**Table 2:** Analysis of Molecular Variance (AMOVA) within species of *Heteropriacanthus* found in different biogeographic regions. NS: not significant. ***: p < 0.001.

		Between regions		Between populations		Within populations
Species	Regions	Variation (%)	Φ_CT_	Variation (%)	Φ_ST_	Variation (%)
*H. fulgens*	Central and East Atlantic	-0.76	-0.008NS	36.70	0.359***	64.06
*H. carolinus*	Indian Ocean, West, Central and East Pacific	28.13	0.281***	2.99	0.311***	68.88

In the Greater Caribbean *H. cruentatus* Φ_ST_ values between populations (except for that between Panama and Venezuela) were small and not significant (Table 1). There was no correlation between Φ_ST_ and the logarithm of geographic distance (r = -0.295, p = 0.77). No AMOVA comparison was possible in *H. cruentatus*, because its distribution does not span oceanic regions. In *H. fulgens* pairwise Φ_ST_ values were small between populations in the central Atlantic, but large and significant within the eastern Atlantic and between populations from Cabo Verde and São Tomé. In AMOVA, however, as most of the variation was within populations and between Cabo Verde and São Tomé, comparisons between east and central Atlantic regions produced an Φ_CT_ value not different from random (Table 2). The correlation of Φ_ST_ and the logarithm of geographic distance was not significant (r = 0.877 p < 0.08).

To further investigate patterns of population structure and gene flow we used MIGRATE-n to test possible models and determine direction of gene flow. Given the general homogeneity between populations within each oceanic region, in these models we considered each region as a single population. In the Atlantic we tested a model of (1) panmixia within the entire Atlantic Ocean *vs*. models in which (2) eastern, central and western Atlantic contain separate populations, and a model (3) in which the populations in the west Atlantic are separate from the combined populations of the central and eastern Atlantic. As would be expected from the phylogenetic separation between Caribbean populations from those in the central and the eastern Atlantic, analysis of the Bayes factors (Table 3) rejected the models of panmixia and the model of independent populations in each region in favor of the hypothesis that the Atlantic is inhabited by two populations, one in the Caribbean, the other occupying the rest of the tropical Atlantic Ocean. The Bayes Factor favoring the two-population model over the three population model is 9.78, which is considered as “moderate evidence” in favor of the former^43^. The Bayes Factor over the panmixia model is 262, or “very strong evidence”. Because MIGRATE-n requires that each population be connected by gene flow to at least one other population, and because the phylogeny shows no shared alleles between *H. cruentatus* and *H. fulgens*, we limited our estimates of genetic exchange to comparisons between the eastern and the central Atlantic. This analysis suggested that the central and eastern Atlantic populations of *H. fulgens* are connected with high and nearly symmetrical gene flow, even though their effective population sizes are small (Table 3).

**Table 3:** Comparison of models of population structure between* Heteropriacanthus *from major regions of the Atlantic Ocean, and estimates of the number of propagules exchanged in each direction per generation (M) and mutation scaled effective population size (Θ) obtained from the best model. Major regions are defined as the eastern, central and western Atlantic. “One population” assumes panmixia in the entire ocean, “two populations” assumes that populatons in the western Atlantic are separate from those in the central and eastern Atlantic, “three populations” assumes that populations in each region exchange genes in both directions. Θ value in each row refers to the first population listed under direction of migration.

Model	Marginal likelihood	Probability
One population	-13986.43	1.34E-114
Two populations	-13724.23	1.00E+00
Three populations	-13734.01	5.65E-05
Direction of migration	M	Θ
Central to East Atl.	319.83	0.08
East to Central Atl.	415.49	0.09

Given the multiple regions in the vast Indo-Pacific, there is a multitude of models that could be tested in MIGRATE-n regarding the direction of gene flow in this ocean. We chose to limit our tests to comparisons of two models that are most likely to reflect reality: Wright’s^40^ island model and Kimura and Weiss’s^41^ stepping stone model. Bayes Factors indicated that the model to which the data fit best in MIGRATE-n was the stepping stone model, according to which neighboring populations exchange genes, but distant populations are isolated from each other (Table 4). The Bayes Factor supporting the stepping stone model was 98.5, considered as “very strong evidence”^43^. The direction of gene flow between oceanic regions was not always symmetrical. Many more propagules arrive in the eastern Pacific from the Central Pacific than travel in the other direction. An asymmetry in the opposite direction seemed to be present between the central and the western Pacific but, given that our only sample in the western Pacific was from Guam, this pattern may not hold for the entire region. Genetic exchange between the Indian Ocean and the western Pacific was high and symmetrical.

**Table 4:** Comparison of models for population structure between* Heteropriacanthus carolinus *from major regions of the Indo-Pacific Ocean, and estimates of the number of propagules exchanged in each direction per generation (M) and mutation scaled effective population size (Θ) obtained from the best model. Major regions are defined as the eastern, central and western Pacific, and the Indian Ocean. “Island model” assumes that populatons in each region exchange genes in both directions with populations in all other regions. “Stepping stone” assumes that genetic exchange is limited to geographically adjacent regions. Θ value in each row refers to the first population listed under direction of migration.

Model	Marginal likelihood	Probability
Island model	-8304.92	1.26E-42
Stepping stone	-8208.44	1.00E+00
Direction of migration	M	Θ
East to Central Pacific	18.00	0.06
Central to East Pacific	432.17	0.07
Central to West Pacific	482.00	0.07
West to Central Pacific	20.83	0.62
West Pacific to Indian Ocean	479.17	0.62
Indian Ocean to West Pacific	481.83	0.06

## Discussion

Our data of concatenated sequences of two mitochondrial and three nuclear genes confirm the conclusions of Fernandez-Silva and Ho^14^ from cytochrome oxidase I and morphology that *Heteropriacanthus* is not a monotypic genus, but includes three allopatric species, *H. carolinus* in the entire Indo-Pacific and *H. cruentatus* as well as *H. fulgens* in the Atlantic. Our data, however, do not corroborate Fernandez-Silva and Ho’s conclusions regarding the ranges of the Atlantic species. They list *H. fulgens* as limited to the northeast region of the Atlantic south to Cabo Verde, but we also found it to be present in São Tomé, Ascension and St. Helena, and (if our single specimen from Brazil is a good indication) also in the southwest Atlantic, without any evidence that it coexists in any of these areas with *H. cruentatus*. Fernandez-Silva and Ho examined the morphology of specimens from the central Atlantic and from Brazil but assigned them to *H. cruentatus*. Morphological differences between *H. cruentatus* and *H. fulgens* are so slight that it is difficult to distinguish between them^11,14^, but it is also possible that the two species coexist in some areas but were missed by our sampling. The reef fish fauna of Brazil contains various other species that also occur in the eastern and central Atlantic^44^, such as *Chromis limbata*^45^*, Aulostomus strigosus*^46^, *Parablennius pilicornis*^47^, *Acanthurus monroviae*^48^, and *Epinephelus marginatus*^48^.

More difficult to explain is the single COI sequence that led Gaither et al.^13^ to propose that propagules of *H. cruentatus* occasionally enter the southwest Indian Ocean. Gaither et al, (at the time that it was still assumed that *H. cruentatus* was the only species in the Atlantic) reported that the sequence of one specimen (GenBank no. KT248776) belonged to *H. cruentatus* on the Indian Ocean side of South Africa. This finding, along with (more equivocal) evidence from three individuals differing from *H. carolinus* in a single base at the S7 intron, led them to conclude that there may be occasional colonization of the southern Indian Ocean from the Atlantic through the Benguela Upwelling and against the usual flow of the Agulhas current. KT248776 was also included in the COI phylogeny of Fernandez Silva and Ho^14^. In their tree, this COI sequence grouped in the clade of *H. cruentatus* instead of *H. fulgens*. We found no genotypes of either of the two Atlantic species at Réunion or the Seychelles, but, more important, no evidence that the range of *H. cruentatus* extends to either the central or the west Atlantic. It is hard to imagine how *H. cruentatus* could have entered the Indian Ocean from the Greater Caribbean without a stepping stone in the central or the eastern Atlantic. As KT248776 was obtained from a museum specimen^14^, it is possible that the collection locality was erroneous, but it is also possible that *H. cruentatus* is, in fact, present in the southeastern Atlantic but we failed to sample it. Denser genetic sampling is needed to determine the correct distributions of the species of *Heteropriacanthus* and answer the question of whether they are, indeed, allopatric, a question that has a bearing on their mode of speciation.

Assuming that *Heteropriacanthus* is composed of three allopatric species, what is their phylogeography? The species distributions are bound by some of the major oceanographic barriers but not by others. *H. cruentatus* and *H. fulgens* are separated from each other by the open ocean between the Caribbean and the central Atlantic^49^ and, possibly, by the outflow of the Orinoco and the Amazon^50^. *H. carolinus* is separated from *H. cruentatus* by the Central American Isthmus^12^ and from *H. fulgens* by the Benguela upwelling^51,52^. Gaither et al.^13^ estimated a coalescence time of the genus as 15 million years ago and, based on that, suggested that the separation the Atlantic and Pacific clades may have been caused by the blockage of the Tethys Sea. However, if the ancestor had a circumglobal distribution, a single barrier would not account for its splitting, as the Central American Isthmus was not in place at that time. As Lessios and Robertson^20^ had also found, the Eastern Pacific Barrier, which constitutes a major and old barrier to most shallow water organisms, has not resulted in speciation in *Heteropriacanthus*, and, according to our findings, neither has the Pleistocene emergence of the Sunda Shelf.

Even though all the “hard” barriers that have caused speciation in other marine organisms have not done the same in *Heteropriacanthus*, their effects are registered in the population genetics of these species. AMOVA (Table 2), pairwise Φ_ST_ statistics (Table 1), the MIGRATE-n coalescent analysis (Table 4), and--to some degree--the distribution of mitochondrial genotypes (Fig. 3B) indicate that there are restrictions to gene flow between the Indian Ocean populations of *H. carolinus* and those of Pacific populations. Our sampling, limited to very distant localities of the two oceans, does not permit conclusions as to the relative contributions to this phenomenon of historical effects caused by the emergence of the Sunda shelf *versus* mere distance. Either way, the significant correlation between genetic and geographic distance in this ocean supports a stepping stone model of genetic exchange and isolation by distance, as does the coalescent analysis of MIGRATE-n. Nevertheless, there is a remarkable degree of connectivity between distant populations of *Heteropriacanthus*, usually seen in species in which adults are strong swimmers, such as tunas^53,54^ and some sharks^55^. How this connectivity is maintained over generations is hard to explain from the life history of this genus. Although *Heteropriacanthus* has a moderately long planktonic larval duration, other reef species with distinctly longer PLD show much more divergence between regions. For example, in the goatfish *Mulloidichthys*, with a PLD of 45-50 days, there are seven extant species, with obvious morphological differences, represented by different mitochondrial DNA clades, and, in several cases, distributed on different sides of biogeographic barriers^56^.

In the Atlantic, populations of *H. cruentatus* spread between the Bahamas and Grenada show little genetic divergence (Table 1). There is higher intraspecific variation in *H. fulgens*, but it does not follow hierarchical population structure between different parts of the ocean (Table 2). MIGRATE-n with populations from each region pooled, also did not support genetic subdivision between the two central and the two eastern Atlantic populations (Table 3). Apparently, the pelagic phase of *H. fulgens* maintains stronger connections along the paths of the currents that traverse the Atlantic^57^ than along the coast of Africa. Four large rivers, the Senegal, the Gambia, the Volta, and the Niger, outflow on the African coast between Cabo Verde and São Tomé. Their effects on connectivity of coastal marine species in the southeast Atlantic has not yet received attention in marine phylogeography. It is conceivable that they are similar to the effects of the outflow of the Amazon and the Orinoco in the southwest Atlantic^50^.

In conclusion, *Heteropriacanthus*, a genus with such slowly evolving external morphology that until recently was thought to consist of a single circumglobal species, when sampled genetically displays a phylogeographic pattern not substantially different from other shallow water genera with similar distributions (review in ref.^6^). Its species are arranged on either side of three of the major biogeographical barriers. Internally, each species shows genetic variation not unexpected for shore fishes with long planktonic phases, but nevertheless indicating a high degree of connectivity for a fish with an adult phase mostly tied to reefs^58^. What aspects of its ecology have provided the selection pressures resulting to low morphological variation is a question well-worth studying.

## Supplementary Information

Supplementary Information.

## Data Availability

Sequences were submitted to GenBank with Accession numbers PX719661-PX719817 for ATPase, PX719232-PX719361 for RAG1, PX719362-PX719507 for RAG2, and PX-719508 PX719660 for TMO.
