# Supplementary material for: Phylogeography of Heteropriacanthus, a circumtropical reef fish with very little morphological variation
Source: Sci Rep. 2026 Apr 11;16:17024. doi: 10.1038/s41598-026-47623-2 (PMC13230789; doi:10.1038/s41598-026-47623-2)

Phylogeography of *Heteropriacanthus*, a circumtropical reef fish with very little morphological variation.

H.A. Lessios, A. Calderón, L. Calderon, L. B. Geyer

Supplementary Information

Table S1. Summary statistics of samples.

Population	N	No. of haplotypes	No. of variable sites	Nucleotide diversity (π)
Atlantic Panama	11	11	255	0.00203
Los Roques, Venezuela	11	11	255	0.00127
Grenada	9	9	260Ta	0.00171
Bahamas	6	6	339	0.00157
Ascension	10	9	104	0.00131
Cabo Verde	10	10	222	0.00611
St. Helena	10	10	280	0.00191
São Tomé	10	10	267	0.00082
Pacific Panama	3	3	67	0.00061
Isla Coco	11	11	225	0.00039
Galapagos	10	10	169	0.00199
Clipperton	7	7	35	0.00070
Revillagigedos	4	4	234	0.00188
Kiritimati	10	10	296	0.00202
Hawaii	9	9	371	0.00115
Easter Island	5	5	277	0.00046
Guam	5	5	75	0.00182
Reunion	10	10	51	0.00073
Seychelles	5	5	56	0.00092

Supplementary Figures.

Figure S1A. First part of Maximum Likelihood haplotype genealogy of RAG1. Nodes with < 48% support have been collapsed. Haplotypes are named according to the localities in which they were encountered.

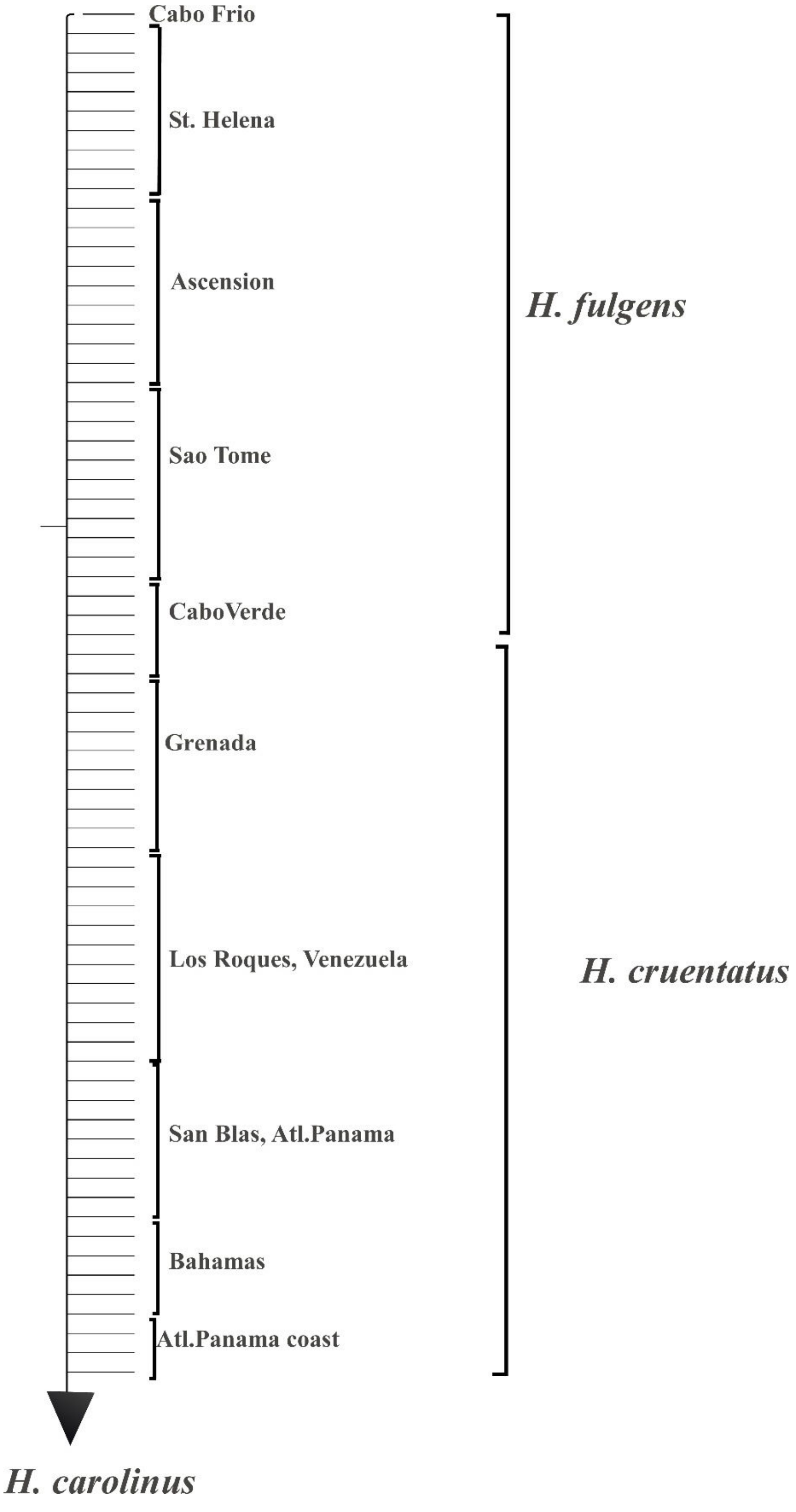

Figure S2B. Second part of Maximum Likelihood genealogy of RAG1.

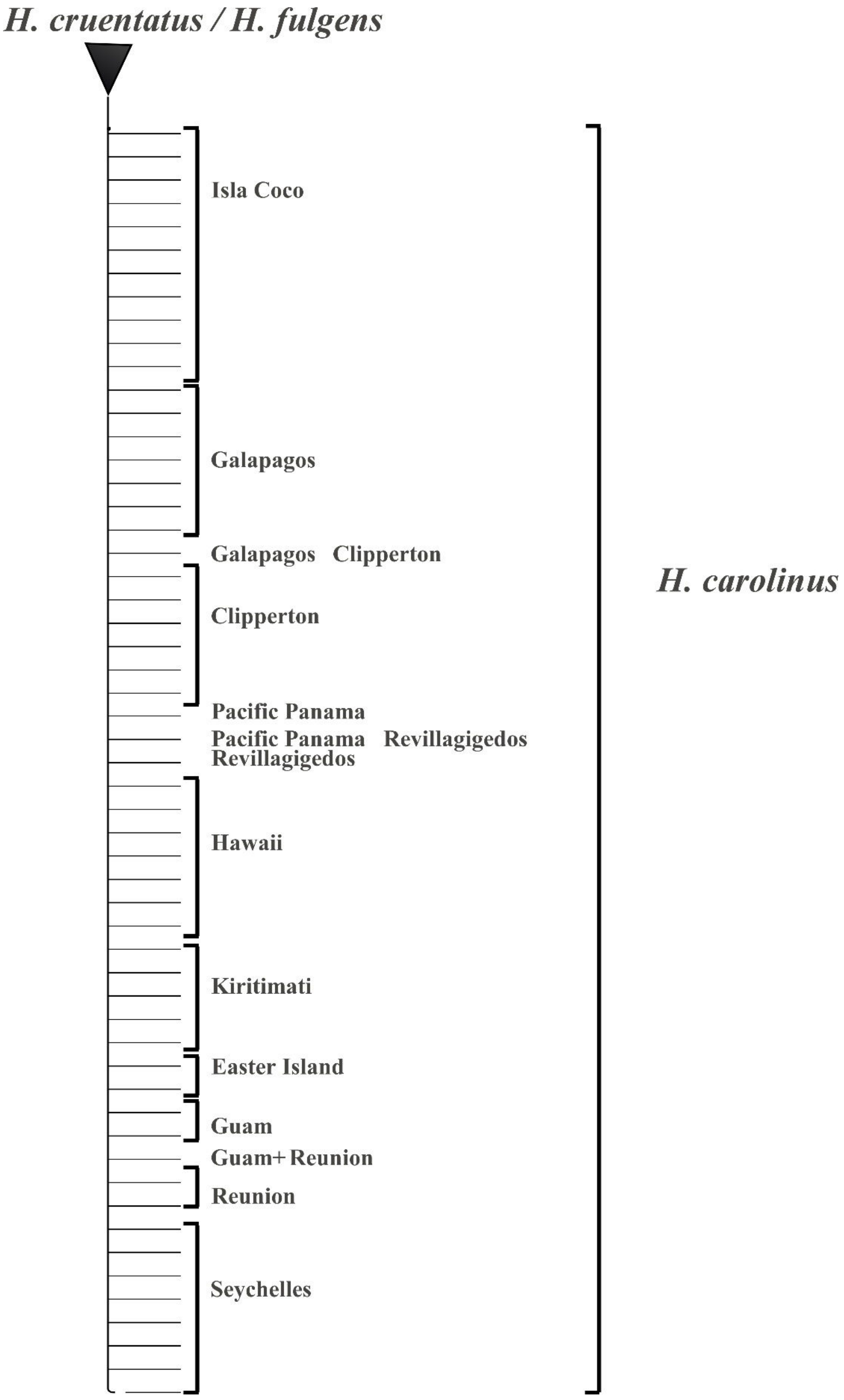

Figure S2A. First part of Maximum Likelihood haplotype genealogy of RAG2. Nodes with <80% support have been collapsed. Haplotypes are named according to the localities in which they were encountered. Numbers in parentheses indicate the number of individuals in a locality bearing the haplotype when >1.

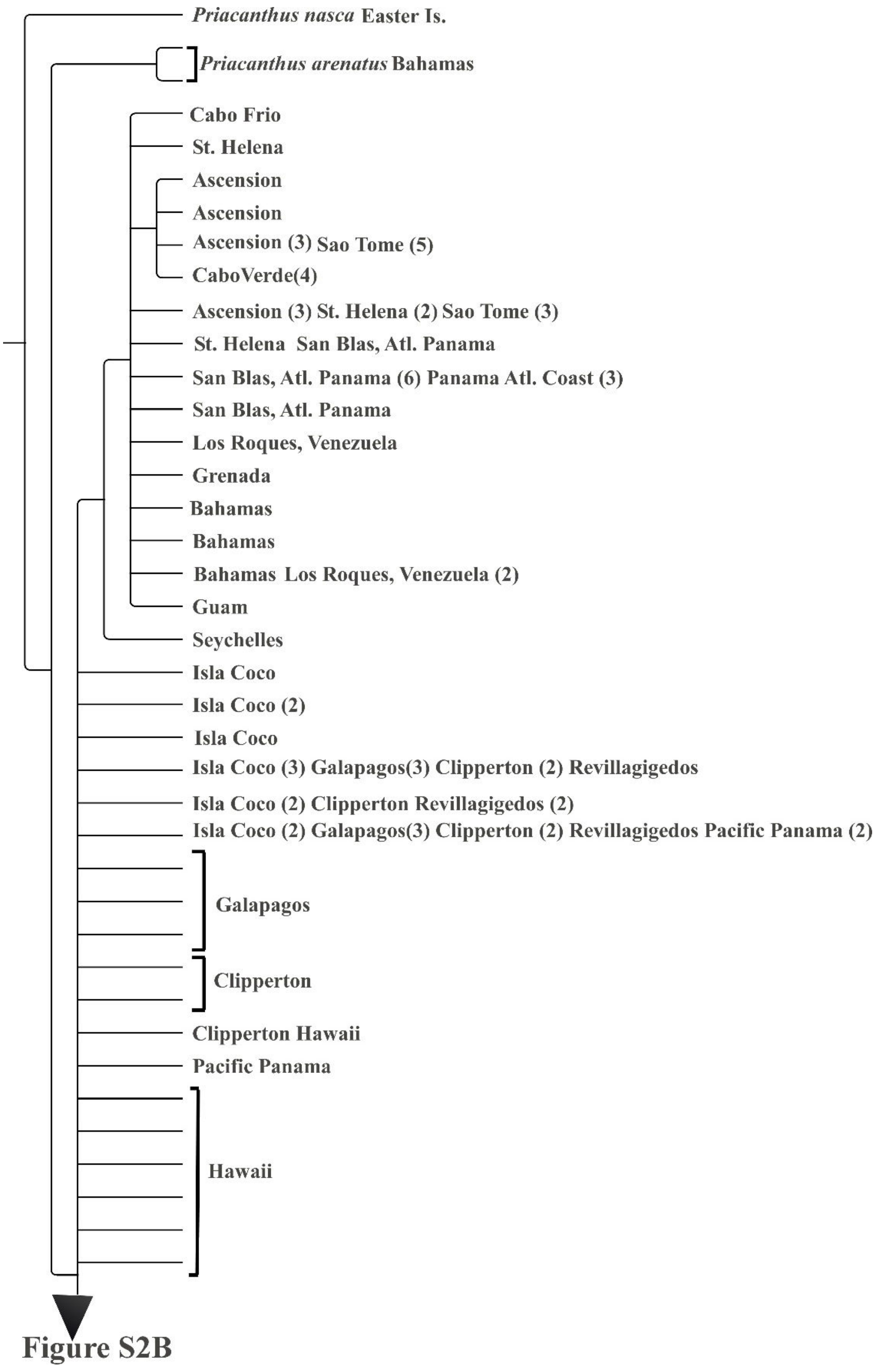

Figure S2B. Second part of Maximum Likelihood haplotype genealogy of RAG2.

Figure S2A

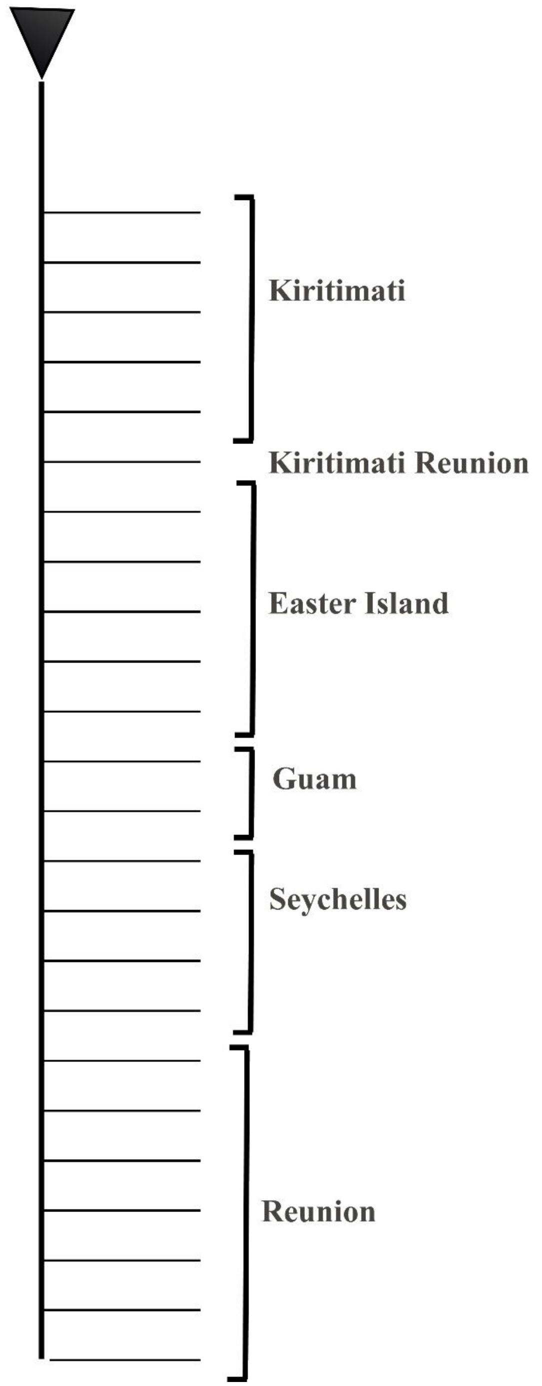

Figure S3A. First part of Maximum Likelihood haplotype genealogy of TMO. Nodes with <80% support have been collapsed. Haplotypes are named according to the localities in which they were encountered. Numbers in parentheses indicate the number of individuals in a locality bearing the haplotype when >1.

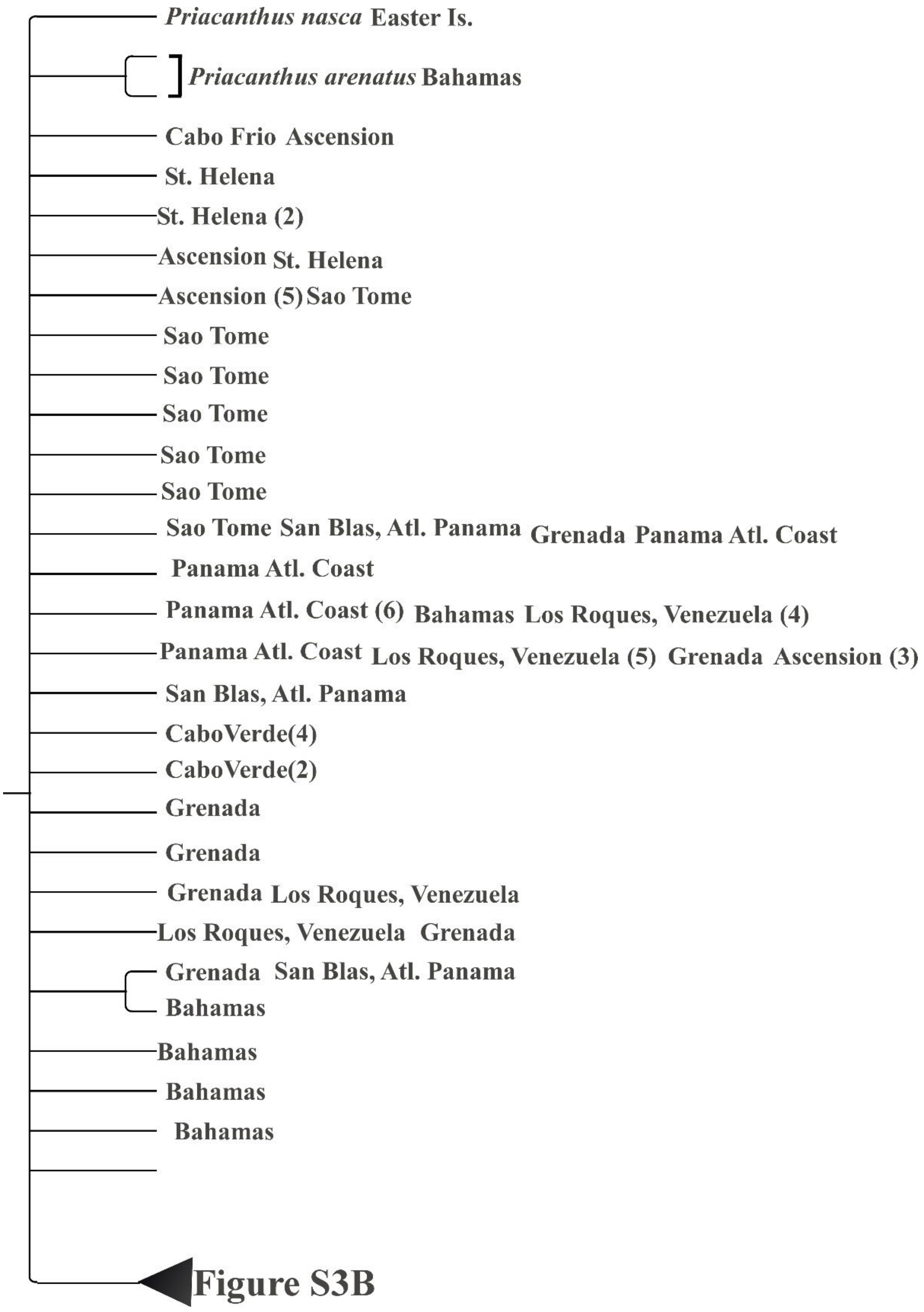

Figure S3B. Second part of Maximum Likelihood haplotype genealogy of TMO.

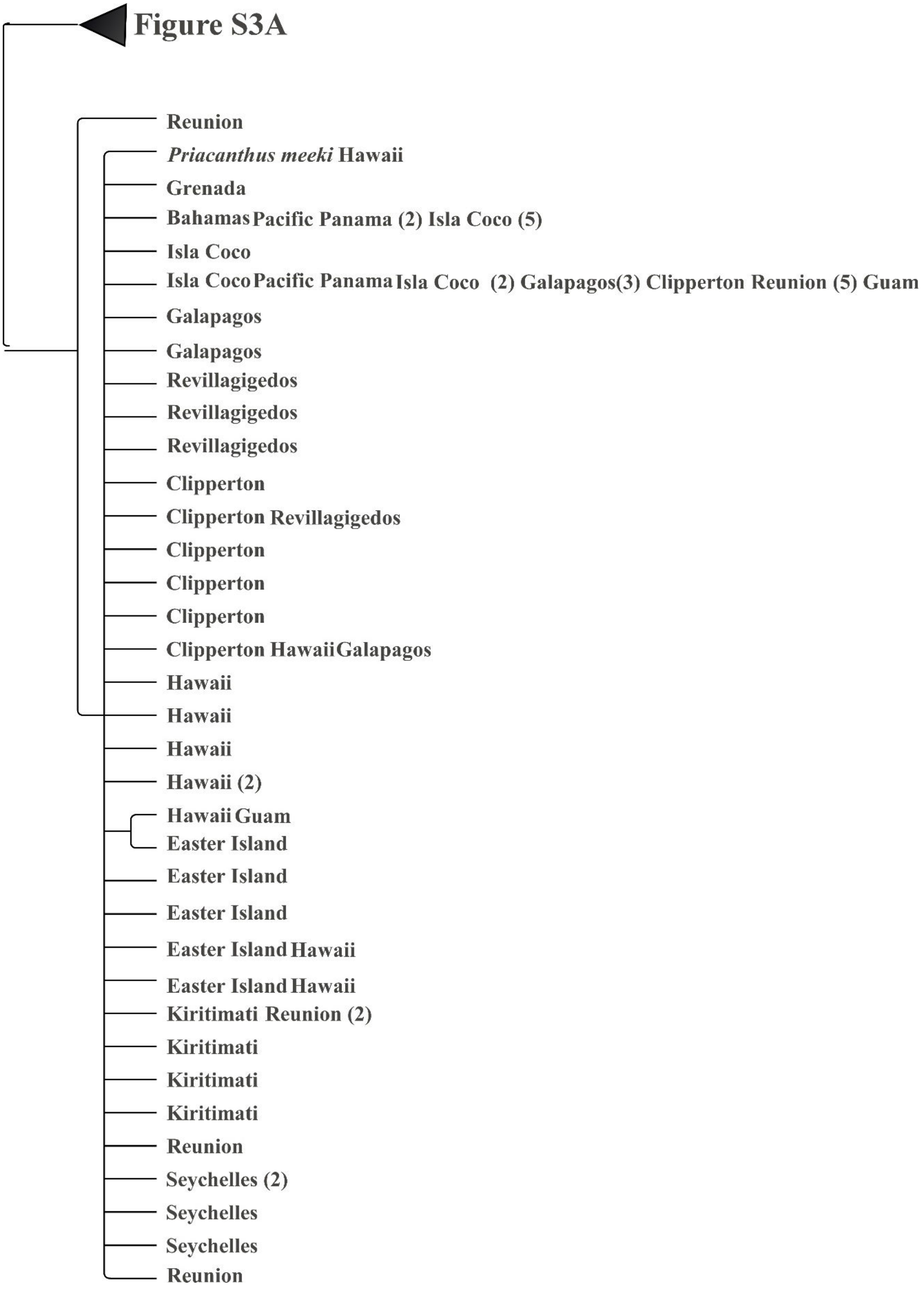

Supplement: Supplementary file 1 — Supplementary Information. [file 41598_2026_47623_MOESM1_ESM.pdf]
